# Wheel-Running Exercise Protects Ovariectomized Mice from Bone Loss via IFN-*γ*-Mediated Suppression of the NF-*κ*B and MAPK Pathways

**DOI:** 10.1155/2022/2030818

**Published:** 2022-05-11

**Authors:** Hao Shen, Jiaheng He, Xuwei Ling, Chang Liu, Yi Wang, Xiongjinfu Zhang, Xu He, Huilin Yang, Mimi Chen, Qin Shi

**Affiliations:** Department of Orthopedics, the First Affiliated Hospital of Soochow University, Orthopedics Institute of Soochow University, Medical College of Soochow University, No. 899, Pinghai Road, Suzhou, Jiangsu 215006, China

## Abstract

Physical exercise is recommended as a preventative approach for osteoporosis; however, the effect of physical exercise on bone mass remains controversial. Additionally, the immune regulation of physical exercise on bone mass remains unclear. To determine whether wheel-running (WR) exercise contributes to improving bone mineral density (BMD) and investigate the involved immune mechanism, ovariectomized (OVX) and sham-operated mice were treated with 8 weeks of WR exercise. The distal femurs of the mice were sequentially scanned, reconstructed, and analyzed using microcomputed tomography and related software to assess BMD and bone microarchitecture. Flow cytometry assays were applied to investigate alterations in immune cells and inflammatory cytokines. *In vitro*, osteoclast differentiation was conducted to determine the effect of IFN-*γ* on osteoclastogenesis and the underlying mechanism. As a result, trabecular parameters were decreased in the OVX mice compared with the sham group. However, WR exercise significantly improved the deterioration in the bone microarchitecture of the OVX mice with an increase of 60.00% in BMD, 55.18% in bone volume, 66.67% in trabecular number, 32.52% in trabecular thickness, and a decrease of 19.44% in trabecular separation. Similarly, WR exercise increased the proportion of CD8^+^ T cells from 7.26 ± 1.71% to 10.23 ± 1.35% in the spleen and from 1.62 ± 0.54% to 2.38 ± 0.43% in the bone marrow of the OVX mice (*P* < 0.05). The expression of IFN-*γ* was also increased in the OVX + WR mice compared with the OVX mice (1.65 ± 0.45% vs. 2.26 ± 0.34%, *P* < 0.05). *In vitro* studies demonstrated an inhibitory effect of IFN-*γ* on osteoclastogenesis in a dose- and time-dependent manner. Meanwhile, the classical NF-*κ*B and MAPK pathways were found to be critical in IFN-*γ*-mediated inhibition of osteoclast differentiation. In conclusion, our study discovered that WR exercise rescued bone loss in the OVX mice in an IFN-*γ*-mediated immunomodulatory manner. After WR exercise, IFN-*γ* expression was restored by activated CD8^+^ T cells, consequently leading to the inhibition of osteoclastogenesis and the recovery from bone loss through the NF-*κ*B and MAPK pathways.

## 1. Introduction

Osteoporosis is an intractable medical challenge characterized by a systemic deterioration of bone mineral density (BMD) and bone microarchitecture, increasing the propensity for fractures [1]. Disruption of the bone remodeling process dominated by bone-resorbing osteoclasts and bone-forming osteoblasts plays a critical role in the pathogenesis of osteoporosis [2, 3]. Postmenopausal women are more prone to developing osteoporosis due to the enhanced bone resorption resulting from estrogen deficiency [4].

To date, the first-line interventions for osteoporosis refer to targeted pharmaceutical agents, combined with general preventive measures such as body weight control, nutrition, and physical exercise [4, 5]. Although physical exercise is widely recommended as a potential strategy to reduce the risk of fracture [6], its effect on bone mass remains controversial, mainly depending on the age of the exerciser and the type of exercise, as well as the volume and intensity [5–8]. The benefits of exercise on the youngers may not hold true for adults or the elders [8–10]. Likewise, not all types of exercise can result in the same beneficial effect on BMD. Even for the same exercise, outcomes could be poles apart due to the volume and intensity. According to McCormack et al., a running volume of 100 kilometers per week contributed to increasing whole-body BMD [11]. Another study performed by Hetland et al. yielded an opposite conclusion that the lumbar spine density was negatively correlated to running distance per week, especially for long-distance runners [12]. As well as the paradoxical results observed in humans, uncertainty exists regarding the effect of running on the bone mass of rodents. Wheel-running (WR) in mice or rats is a widely used model to study exercise in humans [13]. Although the majority of reports revealed a positive link between BMD and WR, the conclusion of null association was also reached [14]. For instance, Takamine et al. reported no significant differences in histological and mechanical properties of bone tissue between the control and WR mice [15]. A negative effect of WR on BMD was also presented by Chen et al. that early life WR exacerbated high fat diet-induced bone loss by accelerating osteoclastogenesis [16]. The problem seems unsolved regarding whether regular running exercise helps to improve BMD.

Physical exercise, seen from another angle, represents a strong inflammatory stimulus and exerts a series of inflammatory events that take place in the immune system [17]. Based on the fact that broad consensus has been achieved concerning definite interactions between the bone and immune system (defined as osteoimmunity or osteoimmunology) [18–20], it is implicit that the immune response to exercise affects bone metabolism to some extent. Diverse mechanisms in the responsiveness of bone to exercise have been theorized previously, involving physical-mechanical stimulation [21], chemical signal transduction [22], and hemodynamic changes [23, 24]. However, it still remains difficult to draw a positive or negative conclusion about the effect of exercise on osteoimmunology, considering the complexity of exercises. In terms of intensity, regular moderate-to-vigorous intensity exercise, rather than the strenuous and exhaustive exercise performed by professional athletes, has been reported to be advantageous to the immune system, while overexercise may cause immunosuppression [25, 26]. For example, the levels of muscle and blood interleukin-6 (IL-6) will increase up to 100-fold after a marathon, which is anti-inflammatory and immunosuppressive and increases the risk of bone loss, while the elevation in IL-6 will return to a basal level in a few hours after moderate-intensity exercise [27]. The net result of the osteoimmune response to exercise mainly depends on the milieu where the response is generated [17]. Unfortunately, the great majority of current knowledge about the milieu is inflammation-related, while alterations of innate or adaptive immunity initiated by physical exercise are of little concern, which is essential for a better understanding of the effect of physical exercise on osteoimmunity.

In the present study, it was assumed that regular running exercise with moderate frequency would improve bone mass by altering the immune response. Based on this hypothesis, we treated the ovariectomized mice with WR exercise to investigate the following: (1) whether the WR exercise contributed to improving BMD, (2) how the immune cells were altered in response to WR exercise, (3) what change took place in cytokines produced by the altered immune cells, and (4) which signaling pathway was involved in the process.

## 2. Materials and Methods

### 2.1. Animal Protocol

**Figure 1:**
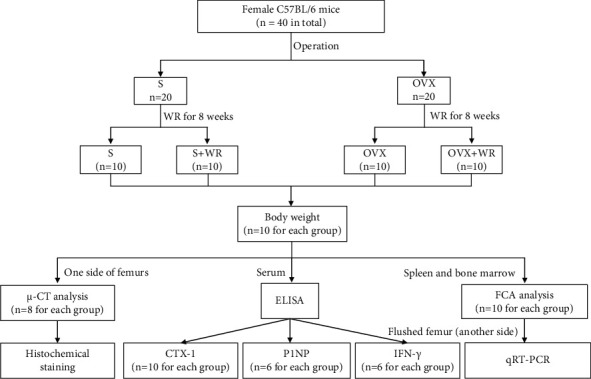
A flowchart of animals used for *in vivo* experiments. S: sham-operated mice; S + WR: sham-operated mice treated with WR exercise; OVX: ovariectomy mice; OVX + WR: OVX mice treated with WR exercise.

Before the study, we conducted a power calculation for sample size according to Charan and Kantharia [28] and the result showed that *n* = 6 for each group was adequate for our research. In case of unexpected loss of samples, we set the sample size as *n* = 10 for each group. As a result, eight-week-old female C57BL/6 mice (*n* = 40 in total) were randomly divided into an ovariectomy (OVX; *n* = 20) group and a sham-operation group (S; *n* = 20). And both the S and OVX groups were subdivided into two subgroups, one for WR exercise (S + WR, OVX + WR; *n* = 10 for each) and the other remaining sedentary (S, OVX; *n* = 10 for each). The randomization was performed systematically according to Lehner [29]. For OVX, bilateral ovaries of the mice were resected under strict aseptic conditions. All surgeries were performed under anesthesia with intraperitoneal (IP) injections of 50 mg sodium pentobarbital per kilogram body weight. Mice were housed under standardized conditions with a 12/12-hour light/dark circadian cycle, an appropriate ambient temperature of approximately 23°C, and free access to chow and water. Body weight was recorded weekly throughout the experiment. The animal protocol was approved by the Animal Care and Use Committee of Soochow University. The flowchart of animals used for *in vivo* experiments is presented in Figure 1.

### 2.2. WR Exercise

WR exercise was conducted two weeks after the operation, utilizing an electric WR instrument (Model YLS-10B, Biowill Co., Shanghai, China) with 8 parallel wheels (wheel width 4 cm, perimeter 60 cm), which was custom designed for mice to prevent from running ad libitum. One week before the formal WR exercise, all the mice were put on the wheels for 1 hour per day to ensure well adaptation. Every assigned mouse performed 30 minutes of WR exercise each time under a rotary speed of 12 revolutions per minute (rpm), with a frequency of twice a day for 8 weeks. At the end of the treatments, all mice were survived and handled for further analyses.

### 2.3. Sample Preparation

Mice were anesthetized at the age of 18 weeks. The blood was collected from the orbital sinus, and serum was separated by centrifugation at 3000 rpm for 15 minutes and stored at 80°C soon afterward until analyses. After exsanguination, the spleens and femurs were isolated aseptically. The spleens were minced using the piston of a 5 mL syringe to generate single-cell suspensions for further flow cytometry assay (FCA). The femurs were fixed in 4% paraformaldehyde (PFA) for 48 hours, transferred into phosphate-buffered saline (PBS), and then scanned by microcomputed tomography (*μ*-CT). The scanned femurs were decalcified in 20% ethylenediaminetetraacetic acid (EDTA; Solarbio, Beijing, China), pH 7.4, for 4 weeks. Subsequently, ethanol gradient dehydration was conducted, and the harvested samples were embedded in paraffin or optimal cutting temperature (OCT) compound (Sakura Finetek, Torrance, USA). Six-*μ*m-thick sections of the femurs were processed for histochemical staining.

### 2.4. *μ*-CT Analysis

Micro-CT (SkyScan1176, Aartselaar, Belgium) was performed under the guideline of Bouxsein [30]. The distal femurs were scanned with the following settings: 50 kV voltage, 500 *μ*A current, exposure time 875 ms, 0.5 mm aluminum filter, 9 *μ*m isotopic resolution, two frames per 0.7° rotation step, and rotation range 180°. The obtained images were three-dimensionally reconstructed using NRecon v1.6 and reoriented using the Data Viewer v1.5 to ensure consistent cross-sectional analysis. A region of interest (ROI) at a total of 100 slices was selected using the CTAn v1.9 software, starting at the location 1 mm proximal to the growth plate and extending proximally. The saved ROI data set was automatically applied to all the samples and trabecular parameters were analyzed using custom processing task lists of the CTAn v1.9. The adaptive threshold was set at 75-255 (lower to upper) for trabecular bone. The estimated BMD was determined based on the linear correction between the Hounsfield Units (HU) and BMD [30]. Trabecular parameters were comprised of BMD, the ratio of trabecular bone volume to total volume (BV/TV), trabecular thickness (Tb. Th), trabecular number (Tb. N), and trabecular separation (Tb. Sp).

### 2.5. Histochemistry and Histomorphometry Analysis

Paraffin sections of the femurs were deparaffinized in xylene, rehydrated in a descending ethanol series (100%, 90%, 80%, and 70%), and stained with hematoxylin and eosin (H&E) or with a tartrate-resistant acid phosphatase (TRAP) staining kit (Sigma-Aldrich, St. Louis, USA). For TRAP staining of osteoclast differentiation, cells were first fixed in 4% PFA for 15 minutes and then stained. TRAP-positive osteoclasts were visualized and captured by a microscope (Zeiss Axio Imager, Oberkochen, Germany). Quantification was determined by the number of TRAP-positive osteoclasts per well.

### 2.6. Enzyme-Linked Immunosorbent Assay (ELISA)

The serum levels of amino-terminal propeptide of type 1 procollagen (P1NP), type 1 collagen C-terminal telopeptide (CTX-1), and interferon-gamma (IFN-*γ*) were measured by ELISA kits (Elabscience, Wuhan, China) according to the manufacturer's instructions. The intra-assay and interassay coefficients of variation were below 6.5% and 7.0% for P1NP [31], 5.5% and 6.0% for CTX-1 [32], and 6.5% and 7.0% for IFN-*γ* [33]. Quantification was measured using a microplate reader (Thermo Fisher Scientific, Waltham, USA) at 450 nm.

### 2.7. FCA

Single-cell suspensions of the spleen and bone marrow (BM) were prepared and then filtered to remove debris. Red blood cells were lysed using ACK lysis buffer (Beyotime, Shanghai, China) to acquire lymphocytes. Lymphocytes were stimulated with 20 ng/mL phorbol 12-myristate 13-acetate (PMA; Sigma-Aldrich, St. Louis, USA), 1 *μ*g/mL ionomycin, and 10 *μ*g/mL brefeldin A (BFA; Sigma-Aldrich, St. Louis, USA) for 5 h at 37°C and then stained for 30 minutes with antimouse CD4-FITC, CD8-PerCP Cy5.5, and IFN-*γ*-APC antibodies (eBioscience, California, USA) at 4°C in the dark. After incubation, the cells were washed with FCA buffer, resuspended in PBS, and assayed with an FCA apparatus (Guava® easyCyte, Merck Millipore, Germany) and the FlowJo v10 software (BD, USA).

### 2.8. Osteoclast Differentiation

Bone marrow-derived macrophages (BMMs) were obtained from the bone marrow of 8-week-old female C57BL/6 mice following a well-established method [34]. After 2 days of stimulation with 30 ng/mL macrophage colony-stimulating factor (M-CSF) for cell attachment, osteoclast differentiation was processed in a 48-well plate (2 × 10^4^ cells/well, 3 technical replicates) in the presence of 30 ng/mL M-CSF, 50 ng/mL receptor activator of nuclear factor-kappa *Β* ligand (RANKL; R&D, Minneapolis, USA), and gradient doses of IFN-*γ* (1, 5, and 10 ng/mL; Peprotech, Suzhou, China) for 6 days. Cytokines were refreshed every 3 days. Another plate was applied to study the time-dependent effect of IFN-*γ* on osteoclast differentiation, in which BMMs were treated with a constant dose of 10 ng/mL IFN-*γ* at different time points, from the beginning (d0) or 3 days (d3) after. Additionally, BMMs were also induced to differentiate in the presence of a neutralizing antibody against the IFN-*γ* receptor (anti-CD119 antibody, 5 *μ*g/mL; Thermo Fisher Scientific, Waltham, USA).

### 2.9. Quantitative Real-Time Polymerase Chain Reaction (qRT-PCR)

The femurs were shattered through a homogenizer (Jinxin, Shanghai, China) after removing bone marrow cells and excessive tissues. Total RNA of the femurs or the seeded cells (BMMs) was extracted using the TRIzol reagent (Beyotime, Shanghai, China) according to the manufacturer's protocols. The purity of RNA was determined by the NanoDrop2000 (Thermo Fisher Scientific, Waltham, USA). Message RNA was reverse transcribed into complementary DNA using a reverse transcriptase kit (TaKaRa, Shiga, Japan), and then, qRT-PCR was performed with the SYBR™ Green PCR Master Mix (Thermo Fisher Scientific, Waltham, USA) on the CFX96™ Real-Time system (Bio-Rad, Hercules, USA). The expression of GAPDH was used as an internal control. Relative expression was calculated according to the comparative Ct (2^−*ΔΔ*Ct^) method. Primers of the targeted genes are listed in Table 1.

### 2.10. Resorption Pit Assay

BMMs were seeded onto an Osteo Assay Plate (OAP; Corning, New York, USA). After attachment, the cells were stimulated with M-CSF, RANKL, and IFN-*γ* as described above. Areas of bone resorption were observed with an inverted microscope, and the total resorption pit was computed with the ImageJ software (Rawak Software Inc., Germany).

### 2.11. Western Blot (WB)

BMMs were seeded in 6-well plates, and cells were cultured with RANKL and IFN-*γ* (10 ng/mL) for 0, 15, 30, and 60 minutes. Protein lysates were made using cell lysis buffer (Beyotime, Shanghai, China) and centrifuged to collect the supernatants for sodium dodecyl sulfate-polyacrylamide gel electrophoresis (SDS-PAGE). Protein concentrations were measured using the BCA Protein Assay Kit (Beyotime, Shanghai, China). WB was conducted on the nitrocellulose membranes. Specific antibodies (anti-actin, MMP-9, CTSK, NFATc1, pi-p65, p65, pi-I*κ*B, I*κ*B, pi-p38, p38, pi-ERK, and ERK; Abcam, Cambridge, UK) were added for incubation at 4°C overnight. Horseradish peroxidase-conjugated secondary antibodies (goat anti-rabbit IgG and goat anti-mouse IgG; Beyotime, Shanghai, China) were incubated for 1 hour at room temperature. The proteins were detected by chemiluminescence, and protein bands were semiquantified by the Image Lab software (Bio-Rad, USA).

### 2.12. Cytoskeleton Staining

BMMs were fixed with 4% PFA and then stained for F-actin by incubating with rhodamine-conjugated phalloidin according to the phalloidin staining kit protocol (Abcam, Cambridge, UK). 4′,6-Diamidino-2-phenylindole (DAPI; Abcam, Cambridge, UK) was used for nuclear staining. Images were captured by a fluorescence microscope (Zeiss Axio Imager, Oberkochen, Germany).

### 2.13. Immunofluorescence (IF) Staining

For IF, nonspecific binding was blocked using immunol staining blocking buffer containing Triton X-100 (Beyotime, Shanghai, China) for 1 hour. Sections were incubated with a rabbit antibody to p65, TRAP, or c-Fos (Abcam, Cambridge, UK) at 4°C overnight, followed by a secondary biotinylated goat antibody to rabbit immunoglobulin G (IgG; Abcam, Cambridge, UK) for 2 h in the dark. DAPI was stained for 10 minutes. All images were acquired using the fluorescence microscope.

### 2.14. Statistical Analysis

All experiments mentioned above were independently replicated three times (biological replicates). The obtained data were analyzed using the GraphPad Prism v8.0 (GraphPad Inc., USA) and IBM SPSS Statistics v26.0 (IBM Corp., USA). The data are presented as the means ± standard deviations (SD). For comparisons between two groups, two-tailed Student's *t*-tests were applied and the effect size is presented with Cohen's *d* value based on Cohen's report [35]. For multiple comparisons among groups, one-way analysis of variance (ANOVA) with Tukey's multiple comparisons tests was used and the effect size is presented with eta squared (*η*^2^) according to Lakens [36]. The significance level was set at *P* < 0.05.

## 3. Results

### 3.1. WR Exercise Decreased Body Weights and Alleviated Bone Loss in OVX Mice

**Figure 2:**
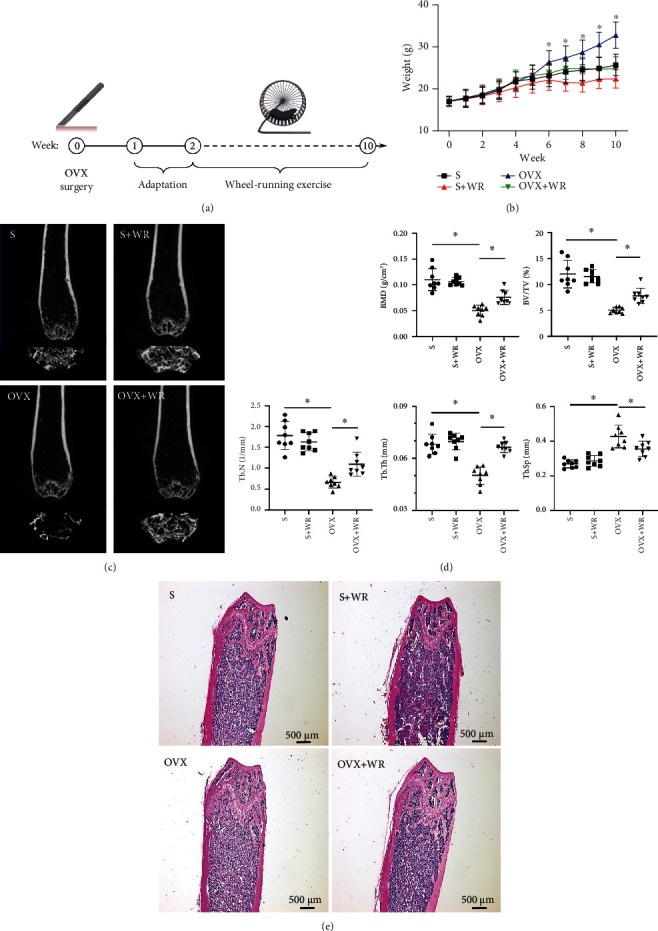
WR exercise decreased body weights and alleviated bone loss in OVX mice. (a) A schematic diagram of the treatments. (b) The body weights of different groups (*n* = 10 mice for each group). (c) Representative *μ*-CT images. (d) *μ*-CT analyses of BMD, BV/TV, Tb. N, Tb. Th, and Tb. Sp (*n* = 8 samples for each group). (e) Representative images of H&E staining. S: sham-operated mice; S + WR: sham-operated mice treated with WR exercise; OVX: ovariectomy mice; OVX + WR: OVX mice treated with WR exercise (^∗^*P* <0.05).

A schematic diagram of the treatments is presented in Figure 2(a). After 8 weeks of WR exercise, the average body weight of the S, S + WR, OVX, and OVX + WR groups was 26.5 ± 3.2 g, 22.8 ± 1.5 g, 32.5 ± 3.9 g, and 25.6 ± 2.1 g, respectively. A significant decrease in the body weights of the exercised mice was observed (Figure 2(b); S + WR vs. S, OVX + WR vs. OVX, *P* < 0.05, *η*^2^ = 0.83). Representative *μ*-CT images (coronal and transaxial sections) of the femurs revealed notable trabecular bone loss induced by OVX, which was evidently alleviated after WR exercise (Figure 2(c)). Further analyses exhibited significant increases in the trabecular parameters of the OVX + WR group compared with the OVX mice, including an increase of 60.00% in BMD (*P* < 0.05, *η*^2^ = 0.77), 55.18% in BV/TV (*P* < 0.05, *η*^2^ = 0.76), 66.67% in Tb. N (*P* < 0.05, *η*^2^ = 0.77), and 32.52% in Tb. Th (*P* < 0.05, *η*^2^ = 0.75), while no significant difference was found in cortical bone (data not shown). Additionally, Tb. Sp, a reflection of the distance between trabeculae, exhibited a decrease of 19.44% in the OVX + WR mice than in the OVX mice (Figure 2(d); *P* < 0.05, *η*^2^ = 0.70). No significant difference in trabecular parameters was revealed between the S and S + WR groups. In accordance with *μ*-CT findings, H&E staining showed increased trabecular bone proximal to the growth plate of the OVX + WR mice compared with that of the OVX mice (Figure 2(e)). These results confirm a protective effect of WR exercise on the bone loss caused by OVX.

### 3.2. WR Exercise Rescued the Bone Loss of OVX Mice by Inhibiting Bone Resorption

**Figure 3:**
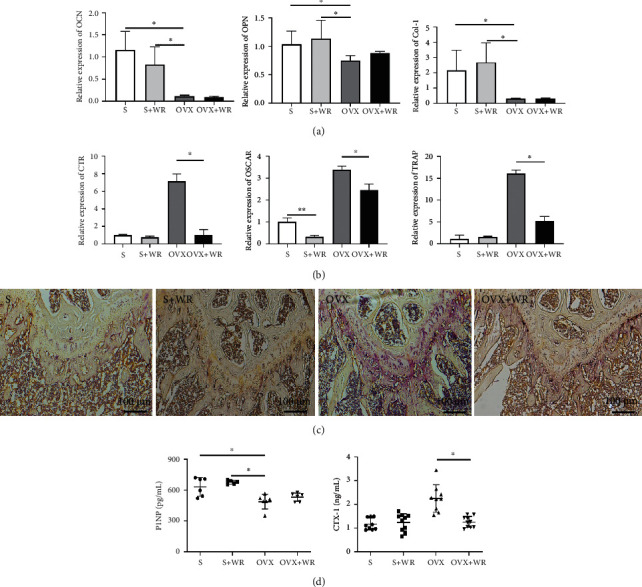
WR exercise inhibited bone resorption in the OVX mice. (a) Relative expression of osteoblast marker genes. (b) Relative expression of osteoclast marker genes. (c) Representative images of TRAP staining. (d) The serum levels of P1NP (*n* = 6 samples for each group) and CTX-1 (*n* = 9 samples for S and OVX, *n* = 10 samples for S + WR, and OVX + WR). S: sham-operated mice; S + WR: sham-operated mice treated with WR exercise; OVX: ovariectomy mice; OVX + WR: OVX mice treated with WR exercise (^∗^*P* < 0.05).

Bone remodeling is dominated by bone-forming osteoblasts and bone-degrading osteoclasts [37]. We next examined whether the protective effect of WR exercise on BMD was caused by increased osteogenesis or decreased bone resorption. The expression of osteoblast marker genes, such as osteocalcin (OCN), osteopontin (OPN), and type I collagen (Col-1), remained unchanged after WR exercise (Figure 3(a)). However, the expression of osteoclast marker genes, including calcitonin receptor (CTR), osteoclast-associated receptor (OSCAR), and TRAP, was statistically downregulated in the OVX + WR mice than the OVX mice (Figure 3(b), a decrease of 6-fold, 1.5-fold, and 3-fold for CTR, OSCAR, and TRAP, respectively). The representative image of TRAP staining in the paraffin section of the OVX + WR mice revealed a markedly reduced TRAP^+^ area in comparison with that of the OVX mice (Figure 3(c)), while no significant change was demonstrated in ALP staining (data not shown). Accordingly, the serum level of CTX-1 in the OVX + WR mice was significantly lower than that in the OVX mice (1.26 ± 0.23 vs. 2.25 ± 0.58 ng/mL, *P* < 0.05, *d* = 2.29), while the level of P1NP remained unchanged (Figure 3(d)). For the sham-operation groups (S, S + WR), little difference was observed. These results indicate that WR exercise protects against bone loss by reducing osteoclastogenesis rather than by increasing osteogenesis.

### 3.3. WR Exercise Increased the Proportion of CD8^+^ T Cells and the Expression of IFN-*γ* in OVX Mice

**Figure 4:**
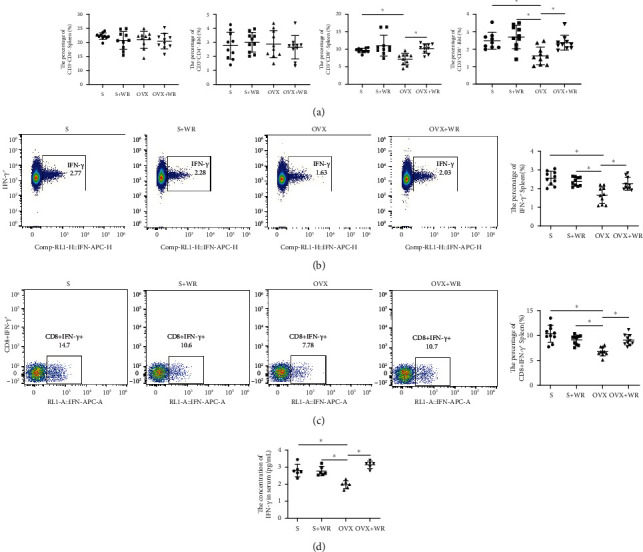
WR exercise increased the proportion of CD8+ T cells and the expression of IFN-*γ* in the OVX mice. (a) The percentage of CD4^+^ and CD8^+^ T cells in the spleen and bone marrow (*n* = 10 samples for each group). (b and c) Representative FCA images and quantitative analyses of the expression of IFN-*γ*^+^ and CD8^+^ IFN-*γ*^+^ cells in the spleen (*n* = 10 samples for each group). (d) The serum level of IFN-*γ* (*n* = 6 samples for each group). S: sham-operated mice; S + WR: sham-operated mice treated with WR exercise; OVX: ovariectomy mice; OVX + WR: OVX mice treated with WR exercise (^∗^*P* < 0.05).

Previous studies have shown that immune cells are highly linked to the bone microenvironment, especially T cells, playing crucial roles in the regulation of bone remodeling [38–40]. We subsequently investigated alterations in the T cell proportions of the four groups. As a result, the proportion of CD8^+^ T cells was significantly decreased in both the spleen and bone marrow of the OVX mice and was enhanced after WR exercise (OVX vs. OVX + WR: 7.26 ± 1.71% vs. 10.23 ± 1.35% in the spleen, *P* < 0.05, *d* = 1.94; 1.62 ± 0.54% vs. 2.38 ± 0.43% in the bone marrow, *P* < 0.05, *d* = 1.57), while CD4^+^ T cells remained unaltered (Figure 4(a)). Among the cytokines produced by T cells, IFN-*γ* plays an indispensable role in osteoporosis [41]. Considering the comparatively lower expression of CD8^+^ T cells in the bone marrow, we further examined the proportions of IFN-*γ* and CD8^+^ IFN-*γ*^+^ cells in the spleen. As presented in Figures 4(b) and 4(c), both the expression of IFN-*γ* (1.65 ± 0.45% vs. 2.26 ± 0.34%, *P* < 0.05, *d* = 1.53) and the proportion of CD8^+^ IFN-*γ*^+^ cells (6.85 ± 0.90% vs. 9.10 ± 1.09%, *P* < 0.05, *d* = 2.24) were significantly increased in the OVX + WR mice in comparison with the OVX group, while no statistical difference was revealed between the S and S + WR groups. The serum level of IFN-*γ* detected by ELISA consolidated the findings (Figure 4(d); OVX vs. OVX + WR: 1.98 ± 0.23 vs. 3.15 ± 0.21 pg/mL, *P* < 0.05, *d* = 5.31). These results show that WR exercise can increase the proportion of CD8^+^ T cells in the OVX mice and subsequently elevate the level of IFN-*γ* produced by CD8^+^ T cells.

### 3.4. IFN-*γ* Inhibited Osteoclast Differentiation and Bone Resorption in a Dose-Dependent Manner

**Figure 5:**
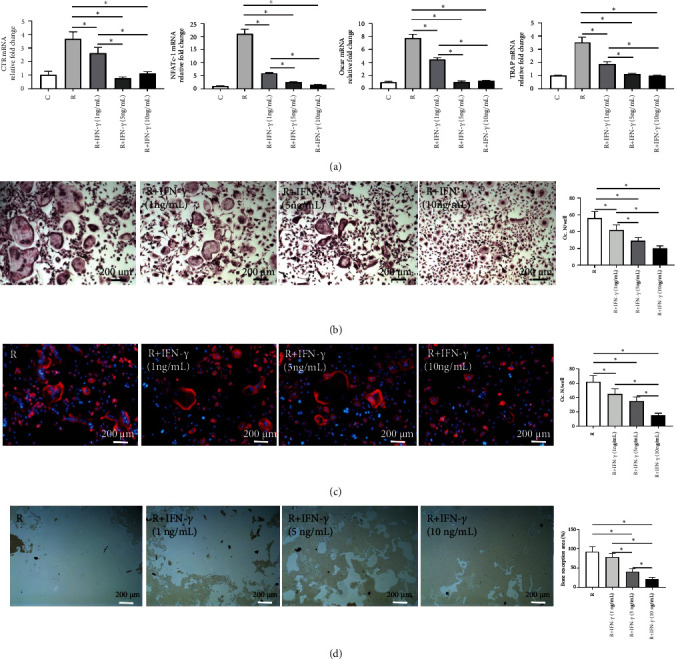
IFN-*γ* inhibited osteoclast differentiation and bone resorption in a dose-dependent manner. (a) Relative expression of osteoclast marker genes after 3 days of osteoclast differentiation. (b and c) Representative images of TRAP staining (b) and F-actin cytoskeleton staining (c) and quantitative analysis of osteoclast number per well (Oc. N/well). (d) Representative images of resorption pit assay and quantitative analysis of bone resorption area (the percentage of the resorbed pit area per total area). C: control (with M-CSF only); R: RANKL (with M-CSF and RANKL) (3 replicates, ^∗^*P* < 0.05).

To investigate the effect of IFN-*γ* on osteoclastogenesis *in vitro*, osteoclast differentiation was induced by cultivating BMMs in the presence of M-CSF and RANKL, accompanied by a gradient dose of IFN-*γ* (0, 1, 5, and 10 ng/mL, respectively). After the BMMs were stimulated for 3 days, qRT-PCR analysis revealed significant dose-dependent suppression of the mRNA expression of osteoclast marker genes, including CTR, OSCAR, TRAP, and nuclear factor of activated T cells 1 (NFATc1; Figure 5(a)). Even at a minimal IFN-*γ* dose of 1 ng/mL tested, osteoclast differentiation was still remarkedly blunted. The mRNA expression levels gradually decreased with IFN-*γ* dosing until reaching a plateau level (5 ng/mL). TRAP and F-actin staining of BMMs differentiated for 6 days confirmed the result in a simple and intuitive way (Figures 5(b) and 5(c)). Furthermore, we examined whether osteoclast differentiation restrained by IFN-*γ* affected bone resorption. The resorption pit assay showed that the proportion of bone resorption area reached 89.52 ± 10.63% in the M-CSF- and RANKL-induced group, while the proportion maximally decreased to 18.43 ± 2.67% with the addition of IFN-*γ* at a dose of 10 ng/mL (Figure 5(d)). Taken together, these results establish that IFN-*γ* exhibits a dose-dependent inhibitory effect on the differentiation of monocytes into osteoclasts and bone resorption.

### 3.5. The Inhibitory Effect of IFN-*γ* on Osteoclastogenesis Was Time-Dependent

**Figure 6:**
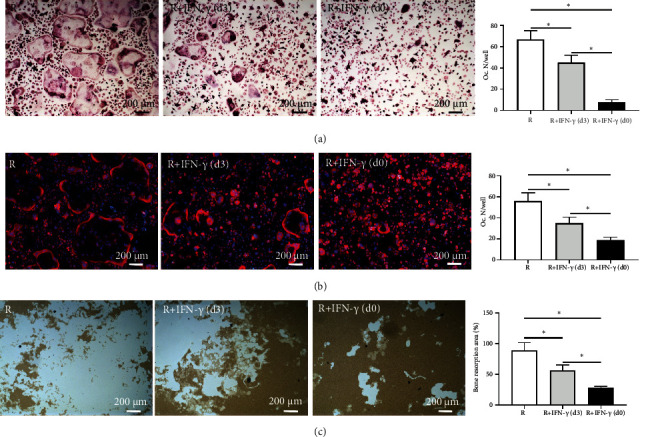
The inhibitory effect of IFN-*γ* on osteoclastogenesis was time-dependent. BMMs were induced to differentiate into osteoclasts, and IFN-*γ* was added at the beginning (d0) or after 3 days (d3). (a and b) Representative images of TRAP staining (a) and F-actin cytoskeleton staining (b) and quantitative analysis of osteoclast number per well (Oc. N/well). (c) Representative images of resorption pit assay and quantitative analysis of bone resorption area (the percentage of the resorbed pit area per total area). C: control (with M-CSF only); R: RANKL (with M-CSF and RANKL) (3 replicates, ^∗^*P* < 0.05).

Previous studies have reported that IFN-*γ* plays dual roles in different stages of osteoclast maturation [41–43]. To elucidate the precise effect of IFN-*γ* on different stages of osteoclastogenesis, we cultured BMMs with M-CSF and RANKL and added IFN-*γ* at an adequate dose of 10 ng/mL at different time points. For BMMs with IFN-*γ* interference from the beginning of induction (d0), osteoclast formation was significantly reduced compared to that of the control group, as demonstrated by TRAP and F-actin staining (Figures 6(a) and 6(b)). Nevertheless, relatively more osteoclasts formed when IFN-*γ* interference was conducted 3 days after induction, indicating that the inhibitory effect of IFN-*γ* was weakened during the late stage of osteoclastogenesis. Our findings were further validated by the bone resorption pit assay (Figure 6(c)). The proportion of bone resorption area was significantly enlarged when the BMMs were pretreated with M-CSF and RANKL for 3 days before IFN-*γ* interference (47.41 ± 6.43%), compared with the group without pretreatment (13.35 ± 2.61%). These results substantiate that the inhibitory effect of IFN-*γ* on osteoclastogenesis is time-dependent and decreases along with the process of osteoclast maturation.

### 3.6. Blockage of IFN-*γ* Signaling Rescued Osteoclast Formation

**Figure 7:**
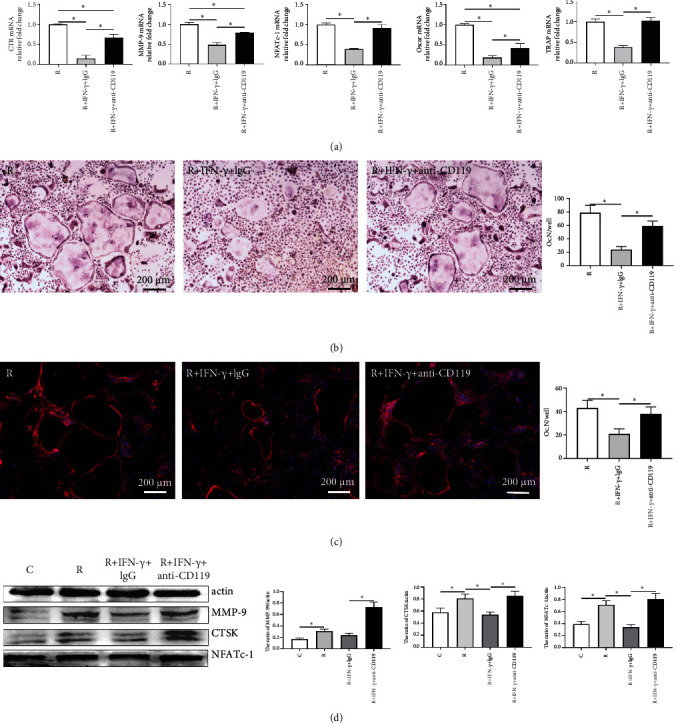
Anti-CD119 antibody counteracted the inhibitory effect of IFN-*γ* on osteoclast differentiation. BMMs were differentiated into osteoclasts with IFN-*γ* (10 ng/mL) and anti-CD119 antibody (5 ng/mL). (a) Relative expression of osteoclast marker genes after 3 days of differentiation. (b and c) Representative images of TRAP staining (b) and F-actin cytoskeleton staining (c) and quantitative analysis of osteoclast number per well (Oc. N/well). (d) The expression and quantitative analyses of MMP-9, CTSK, and NFATc1 in nuclear extracts of BMMs by western blot performed after 6 days. C: control (with M-CSF only); R: RANKL (with M-CSF and RANKL) (3 replicates, ^∗^*P* < 0.05).

To further authenticate the inhibitory effect of IFN-*γ* on osteoclastogenesis, we cultured BMMs in the presence of differentiation stimulating factors and IFN-*γ* or anti-CD119 antibody. qRT-PCR analysis revealed that the expression of osteoclast-related genes was partly recovered by the anti-CD119 antibody (Figure 7(a)). TRAP staining on day 6 showed that blockage of IFN-*γ* signaling resulted in a substantially increased number of osteoclasts in comparison with the IgG control group (Figure 7(b)). Similar findings were obtained by F-actin staining (Figure 7(c)). In terms of protein expression, WB analysis showed the ability of BMMs to differentiate into osteoclasts was recovered by demonstrating increased expression of osteoclast marker proteins including cathepsin K (CTSK), matrix metalloproteinase-9 (MMP-9), and NFATc1 in the anti-CD119 group compared with the IgG control group (Figure 7(d)). These data confirm that IFN-*γ* is a direct inhibitor of osteoclast formation.

### 3.7. IFN-*γ* Inhibited Osteoclastogenesis by Suppressing RANKL-Mediated Activation of the NF-*κ*B and MAPK Pathways

**Figure 8:**
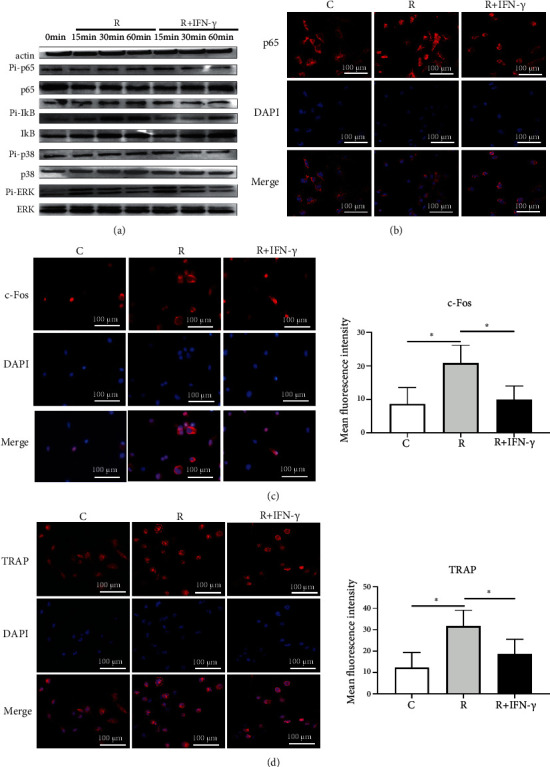
IFN-*γ* inhibited osteoclastogenesis by suppressing RANKL-mediated activation of the NF-*κ*B and MAPK pathways. BMMs were differentiated into osteoclasts with IFN-*γ* (10 ng/mL). (a) The expression of p65, Pi-p65, I*κ*B, Pi-I*κ*B, p38, Pi-p38, ERK, and Pi-ERK in nuclear extracts of BMMs by western blot performed after 6 days. (b) Representative images of p65 translocation in the differentiated BMMs by immunocytochemical staining performed after 30 minutes. (c and d) Representative images and quantitative analyses of c-Fos (c) and TRAP (d) in differentiated BMMs by immunocytochemical staining performed after 2 days. C: control (with M-CSF only); R: RANKL (with M-CSF and RANKL) (3 replicates, ^∗^*P* < 0.05).

To recognize the downstream mechanism of IFN-*γ* in osteoclastogenesis, we cultured BMMs in the differentiation medium supplemented with 10 ng/mL IFN-*γ* for 15, 30, and 60 minutes. WB showed that IFN-*γ* significantly abated RANKL-induced phosphorylation (Pi) of key proteins in nuclear factor kappa-B (NF-*κ*B) and mitogen-activated protein kinase (MAPK) signaling pathways (Figure 8(a)), including p65 kinase, inhibitor of NF-*κ*B (I*κ*B), p38 kinase, and extracellular regulated protein kinase (ERK). Nuclear translocation is necessary for the regulation of protein expression by the NF-*κ*B transcription factor family. Representative NF-*κ*B translocation to the nucleus was presented by immunocytochemical staining of p65, a major component of the NF-*κ*B transcription factor family. Obvious RANKL-induced p65 translocation from the cytoplasm to the nucleus was observed compared to the control group (with M-CSF only), while remarkably impaired nuclear translocation was monitored in the presence of IFN-*γ* (Figure 8(b)). Moreover, the expression of c-Fos, a vital transcription factor regulated by the MAPK pathway, was investigated. Immunofluorescence staining indicated a striking decline in c-Fos expression under IFN-*γ* treatment in comparison with the RANKL-induced group (Figure 8(c)). Additionally, the proportion of TRAP^+^ staining area dropped from 30.43 ± 4.75% to 18.62 ± 2.15% after IFN-*γ* treatment (Figure 8(d)). Collectively, these results demonstrate that the inhibitory effect of IFN-*γ* on osteoclastogenesis is exerted by suppressing RANKL-mediated activation of the NF-*κ*B and MAPK signaling pathways.

## 4. Discussion

**Figure 9:**
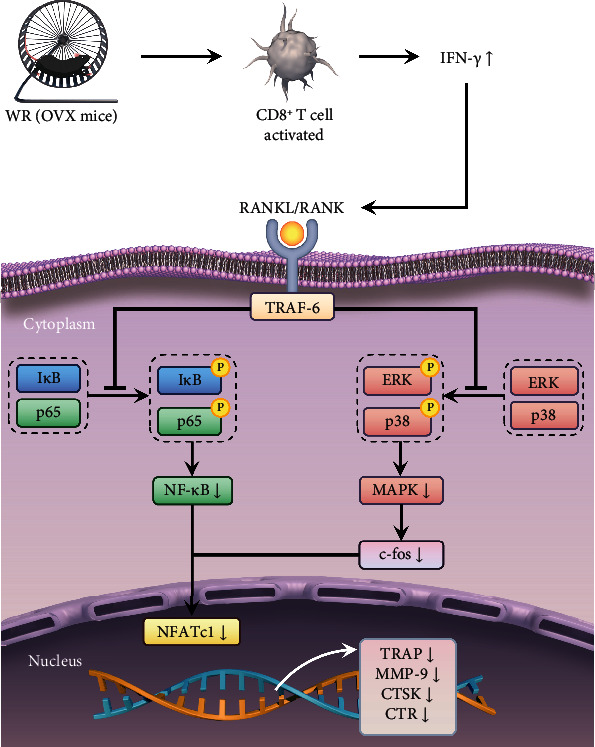
Summary of the proposed mechanism responsible for the recovered bone loss of OVX mice after WR exercise. In OVX mice, WR exercise activates CD8^+^ T cells to produce the inflammatory cytokine IFN-*γ*, which inhibits osteoclastogenesis and bone resorption through the NF-*κ*B and MAPK signaling pathways. The suppression of I*κ*B and p65 phosphorylation in the NF-*κ*B pathway, as well as the suppression of ERK and p38 phosphorylation in the MAPK pathway, triggers a decreased level of NFATc1. As a result, the expression of osteoclast marker genes is downregulated.

In this study, we disclosed several novel discoveries on the immune pathogenesis of osteoporosis and the antiosteoporotic effect of WR exercise. The main results of *in vivo* experiments are organized in Table 2. We showed a significantly decreased level of CD8^+^ T cells in the OVX mice. Subsequent lower expression of IFN-*γ* diminished its inhibitory effect on osteoclastogenesis, resulting in elevated bone resorption. We further demonstrated reversed alterations in CD8^+^ T cells and IFN-*γ*, as well as recovered bone mass, by performing regular WR exercise on OVX mice. The RANKL-mediated NF-*κ*B and MAPK pathways were revealed to be critical in the antiosteoporotic effect of WR exercise. In a word, we discovered that WR exercise protects against osteoporosis by activating CD8^+^ T cells to release IFN-*γ*, which inhibits osteoclastogenesis through the NF-*κ*B and MAPK pathways (Figure 9).

Although physical exercise is widely recognized as an effective approach for eliciting a positive skeletal response, exercise recommendations for the prevention or management of osteoporosis should be specific and personalized, considering that the impact of exercise on bone is modality-, volume-, and intensity-dependent [44]. By far, moderate to intense exercise has been proven osteogenic [45, 46], but little consensus has been achieved when it refers to running, a type of intense exercise according to Brian et al. [47]. In the current study, BMD and bone microarchitecture of the OVX mice were decreased compared with the S group and were improved after 8 weeks of regular WR exercise. Specifically, the deterioration in trabecular bone parameters caused by OVX was rescued by WR exercise with an increase of 60.00% in BMD, 55.18% in BV/TV, 66.67% in Tb. N, and 32.52% in Tb. Th, while no significant cortical bone change was demonstrated. Correspondingly, the elevation in bone turnover marker CTX-1 for osteoclastic activity caused by OVX was decreased to a normal level after WR, while the downregulated level of bone turnover marker P1NP for osteoblastic activity caused by OVX remained unchanged after WR. The results indicated that WR exercise played a preventive role in the development of osteoporosis by inhibiting bone resorption rather than promoting bone formation, consistent with previous findings by Li et al. [45], who performed 17 weeks of treadmill exercise on ovariectomized rats. Contrary to expectations, our study did not detect any significant difference in bone microarchitecture between the two groups without OVX (S and S + WR). This may be explained by the fact that nonprogressive WR exercise with a certain intensity is inadequate to break through the required threshold to generate an adaptive skeletal response at a physiological state. For the OVX mice with relatively lower BMD, a greater skeletal response will occur according to Winters-Stone et al. [48]. Another possible explanation refers to skeletal muscle power. It has been confirmed that muscle fiber atrophy takes place in the presence of osteopenia or osteoporosis [49]. As a result, higher strain rates on bone induced by muscle contractions are registered to optimize bone health in the osteoporosis group under the same stimulus.

All the mechanisms mentioned above are related to physical-mechanical stimulations. Chemical signal transduction is also an indispensable part of the responsiveness of bone to exercise. It has been clear that cells involved in bone metabolism and the immune response both share the microenvironment and interact to affect the bone remodeling process [50]. In particular, T cells, B cells, and inflammatory cytokines are important regulatory factors in the interactions of bone-immune systems, among which the role of T cells is pivotal [38]. Strikingly, not all T cells are functional in bone metabolism in any situation. In our study, only the expression of CD8^+^ T cells was statistically different both in the spleen and the bone marrow, while the level of CD4^+^ T cells remained unchanged after treatment with OVX and/or WR. Considering the variety of T cell subpopulations, further investigations remain to be conducted. Serving as an inflammatory stimulus, WR exercise elicited an increase in the number of CD8^+^ T cells in OVX mice rather than in those without OVX. The difference may be explained by the link between estrogen deficiency caused by OVX and T cell-dependent inflammation, which has been unraveled recently by Cline-Smith et al. [51]. They confirmed that the silenced expression of IL-17A and TNF induced by T cell-specific ablation of IL15RA led to bone loss after OVX. Similar to IL-17A and TNF, multiple other cytokines produced by T cells play essential roles in bone metabolism. IFN-*γ*, an inflammatory cytokine with extensive immunomodulatory effects, has been reported to have miscellaneous impacts on osteoclastogenesis and osteoblastogenesis, which may be linked to different doses and exposure times of IFN-*γ*, as well as the specific stage of cell differentiation [41]. In the current study, the inhibitory effect of IFN-*γ* on osteoclastogenesis was thoroughly evaluated in a dose- or time-dependent manner. Our results revealed that osteoclastogenesis was negatively correlated with the dose and exposure time of IFN-*γ*.

Many signaling pathways are involved in IFN-*γ*-mediated osteoimmunity, among which the classical pathways depending on RANKL-RANK signaling are essential for osteoclast differentiation [41, 52]. After RANKL binds to RANK, a preliminary step in downstream signaling is initiated with the recruitment of an adaptor protein, tumor necrosis factor receptor-associated factor 6 (TRAF6), leading to the activation of NF-*κ*B and MAPK [52, 53]. The canonical NF-*κ*B signaling pathway comprises a number of activation steps as well as a family of transcription factors [54]. I*κ*Bs are inhibitory NF-*κ*B proteins participating in the proteasomal degradation process of the NF-*κ*B signaling pathway. The current results revealed that in the presence of IFN-*γ*, the phosphorylation of I*κ*Bs was inhibited, resulting in a decreased release of phosphorylated p65 (a member of the transcription factor family, also known as RelA). As reported, the inhibition of the NF-*κ*B signaling pathway prevented osteoporotic bone loss induced by OVX [55], which was consistent with our results. Our study also indicated that the NF-*κ*B signaling pathway was not the only pathway involved in the IFN-*γ*-induced inhibition of osteoclast differentiation, since the phosphorylation of ERK and p38 signaling in the MAPK pathway was also downregulated. The suppression of the NF-*κ*B and MAPK signaling pathways reduced the expression of c-Fos and triggered a decreased level of NFATc1, a terminal regulator of osteoclast differentiation. The loss of c-Fos or NFATc1 results in severe osteopetrosis in mice [56]. So, it is reasonable that the suppression of the NF-*κ*B and MAPK signaling pathways contributes to considerable bone mass recovery in our study. Although it may provide a reasonable account for WR exercise-induced bone mass recovery in OVX mice by investigating IFN-*γ*-mediated signal transduction pathway changes *in vitro*, no direct evidence was available for the independence between WR exercise and bone mass of the control mice. Thus, more in-depth experiments involving immune cells and inflammatory cytokines are urgently needed to elucidate the relationship between WR exercise and bone mass under physiological conditions.

Several limitations to our research should be considered. First, due to the complexity of running exercise, more animal groups should be included involving different durations, volumes, and intensities of WR exercise. Second, the evaluation of bone compositions should be site-specific. In addition to the femurs, the vertebrae are also of great importance in the quantification of BMD, which is not reflected in our study. Moreover, although IFN-*γ* was identified as a key cytokine in the antiosteoporotic effect of WR exercise, all the IFN-*γ*-related experiments were carried out *in vitro*. To consolidate the findings, *in vivo* investigations using exogenous IFN-*γ* or anti-IFN-*γ* antibodies should be advanced.

## 5. Conclusion

We demonstrated that WR exercise rescued bone loss in the OVX mice in an IFN-*γ*-mediated immunomodulatory manner. The release of IFN-*γ* was increased by activated CD8^+^ T cells resulting from WR exercise, consequently leading to the inhibition of osteoclastogenesis, which was due to the suppression of the NF-*κ*B and MAPK pathways. The findings contribute to a better understanding of the regulatory mechanisms of IFN-*γ* on osteoclast activities and shed new light on the immune relationship between running exercise and osteoporosis.

## Figures and Tables

**Table 1:** The sequence of the primer.

Gene name	Primer sequence
GAPDH	Forward 5′-GCACAGTCAAGGCCGAGAAT-3′
	Reverse 5′-GCCTTCTCCATGGTGGTGAA-3′

CTR	Forward 5′-CCTCTTGCCCTTGGGTGCTATC-3′
	Reverse 5′-CTGGGGAGTAAAGAGGGGTATGG-3′

CTSK	Forward 5′-ATGTGGGTGTTCAAGTTTC-3′
	Reverse 5′-TCAATGCCTCCGTTCT-3′

NFATc1	Forward 5′-TGGGAGATGGAAGCAAAGAC-3′
	Reverse 5′-ATAGAAACTGACTTGGACGGG-3′

MMP-9	Forward 5′-GCCGACTTTTGTGGTCTTCC-3′
	Reverse 5′-GGTACAAGTATGCCTCTGCCA-3′

Oscar	Forward 5′-GTTTGGGGCTGGCAGGAATGGT-3′
	Reverse 5′-GAGGTGGGGAGCCGGAAATAAGG-3′

TRAP	Forward 5′-AGACCCAATGCCACCC-3′
	Reverse 5′-GGACCTCCAAGTTCTTATC-3′

**Table 2:** Main results of *in vivo* experiments.

	Groups				*P* value		
Parameters	*S*	*S* + WR	OVX	OVX + WR	OVX vs. *S*	*S* + WR vs. *S*	OVX + WR vs. OVX
Body weight (g)	26.5 ± 3.2	22.8 ± 1.5	32.5 ± 3.9	25.6 ± 2.1	< 0.05	< 0.05	< 0.05
Trabecular bone							
BMD (g/cm^3^)	0.11 ± 0.02	0.11 ± 0.01	0.05 ± 0.01	0.08 ± 0.01	< 0.05	> 0.05	< 0.05
BV/TV (%)	11.98 ± 2.67	11.51 ± 1.28	5.02 ± 0.55	7.79 ± 1.47	< 0.05	> 0.05	< 0.05
Tb. N (1/mm)	1.78 ± 0.34	1.63 ± 0.22	0.66 ± 0.14	1.10 ± 0.29	< 0.05	> 0.05	< 0.05
Tb. Th (um)	68.07 ± 5.74	69.58 ± 4.73	50.09 ± 5.18	66.38 ± 3.10	< 0.05	> 0.05	< 0.05
Tb. Sp (mm)	0.27 ± 0.02	0.29 ± 0.03	0.43 ± 0.07	0.36 ± 0.04	< 0.05	> 0.05	< 0.05
Serum P1NP level (pg/mL)	631.4 ± 90.6	676.6 ± 19.1	487.6 ± 72.2	531.02 ± 40.4	< 0.05	> 0.05	> 0.05
Serum CTX-1 level (ng/mL)	1.23 ± 0.25	1.25 ± 0.37	2.25 ± 0.58	1.26 ± 0.23	< 0.05	> 0.05	< 0.05
CD4^+^ T in spleen (%)	17.22 ± 1.17	15.73 ± 3.39	15.91 ± 3.01	15.38 ± 2.73	> 0.05	> 0.05	> 0.05
CD4^+^ T in BM (%)	2.76 ± 0.96	3.01 ± 0.7	2.87 ± 0.97	2.65 ± 0.83	> 0.05	> 0.05	> 0.05
CD8^+^ T in spleen (%)	9.67 ± 0.64	11.04 ± 2.97	7.26 ± 1.71	10.23 ± 1.35	< 0.05	> 0.05	< 0.05
CD8^+^ T in BM (%)	2.49 ± 0.49	2.70 ± 0.66	1.62 ± 0.54	2.38 ± 0.43	< 0.05	> 0.05	< 0.05
IFN-*γ*^+^ in spleen (%)	2.56 ± 0.37	2.37 ± 0.25	1.65 ± 0.45	2.26 ± 0.34	< 0.05	> 0.05	< 0.05
CD8^+^ IFN-*γ*^+^ in spleen (%)	10.37 ± 1.71	9.13 ± 0.97	6.85 ± 0.90	9.10 ± 1.09	< 0.05	> 0.05	< 0.05
Serum IFN-*γ* level (pg/mL)	2.81 ± 0.36	2.77 ± 0.27	1.98 ± 0.23	3.15 ± 0.21	< 0.05	> 0.05	< 0.05

## Data Availability

Data in this manuscript are available from the corresponding author on reasonable request.
